# Evaluation of methods for amplification of picogram amounts of total RNA for whole genome expression profiling

**DOI:** 10.1186/1471-2164-10-246

**Published:** 2009-05-26

**Authors:** Mathieu Clément-Ziza, David Gentien, Stanislas Lyonnet, Jean-Paul Thiery, Claude Besmond, Charles Decraene

**Affiliations:** 1 INSERM U781, Université Paris Descartes, Faculté de Médecine, Hôpital Necker-Enfants Malades, 149 rue de Sèvres, F-75015, Paris, France; 2 Institut Curie, Département de Transfert, 26 rue d'ULM, F-75248, Paris cedex 05, France; 3 IMCB Proteos, 61 Biopolis Drive, 138673, Singapore; 4 Institut Curie, Centre de Recherche, 26 rue d'ULM, F-75248, Paris cedex 05, France; 5 CNRS, UMR144, 26 rue d'ULM, F-75248, Paris cedex 05, France

## Abstract

**Background:**

For more than a decade, microarrays have been a powerful and widely used tool to explore the transcriptome of biological systems. However, the amount of biological material from cell sorting or laser capture microdissection is much too small to perform microarray studies. To address this issue, RNA amplification methods have been developed to generate sufficient targets from picogram amounts of total RNA to perform microarray hybridisation.

**Results:**

In this study, four commercial protocols for amplification of picograms amounts of input RNA for microarray expression profiling were evaluated and compared. The quantitative and qualitative performances of the methods were assessed. Microarrays were hybridised with the amplified targets and the amplification protocols were compared with respect to the quality of expression profiles, reproducibility within a concentration range of input RNA, and sensitivity. The results demonstrate significant differences between these four methods.

**Conclusion:**

In our hands, the WT-Ovation pico system proposed by Nugen appears to be the most suitable for RNA amplification. This comparative study will be useful to scientists needing to choose an amplification method to carry out microarray experiments involving samples comprising only a few cells and generating picogram amounts of RNA.

## Background

Gene expression profiling using microarray technology is a powerful method to investigate the phenotype of complex biological systems [1]. Over the last decade, developments by academic and private sectors have improved the reproducibility, standardisation and accuracy, and have decreased the cost of this technology. However, the main drawback of this technology was the large amount of input RNA needed to carry out microarray experiments, preventing expression analysis on small samples. This problem has been largely overcome thanks to RNA amplification methods. Standard target preparation protocols now allow large-scale gene expression profiling to be performed from nanogram quantities of input RNA. However, cell selection technologies that allow the isolation of 1–100 cells such as cell-sorting methods [2] or laser capture microdissection (LCM) [3-5], have driven the development for further reduction. Only a few protocols are designed for the amplification of picogram amounts of input RNA and allow the potential investigation of the transcriptome of few cells isolated by cell-sorting techniques or LCM.

The most routinely used amplification method is based on linear amplification by *in vitro *transcription (IVT) of a cDNA template into complementary RNA (cRNA), using T7 RNA polymerase [6-8]. Several protocols based on this technique have been developed and are commonly used to perform gene expression profiling experiments using microarrays [6,9-20]. Recently, a new RNA amplification system based on the linear isothermal amplification of double-stranded cDNA that encompasses a unique RNA/DNA heteroduplex at one end using the RNA-dependent DNA polymerase activity has been developed [21]. This technique has been used to perform gene expression profiling experiments [22-29].

We assume that the purification of total RNA from cultured cells or tissue after isolation using these methods can be largely method-, cell- or tissue-dependent. In this study, to minimise this source of error and to focus on the amplification method, a commercial source of total RNA, i.e. human universal RNA, was used. We compared four commercial RNA amplification protocols to the standard target labelling procedure proposed by Affymetrix in side-by-side experiments to evaluate the most suitable method to perform gene expression profiling from picogram amounts of input RNA on Affymetrix GeneChip microarrays. The following amplification protocols were compared: i) Arcturus RiboAmp™ system, ii) Ambion MessageAmp™, iii) Epicentre TargetAmp™, and iv) Nugen WT-Amplification™ pico system. Protocols proposed by Arcturus, Ambion and Epicentre are adapted from the IVT method first described by Eberwine et al. [6-8]. The system proposed by Nugen is based on the RNA-dependent DNA polymerase activity [21]. Although amplification methods have already been evaluated elsewhere [15,24,26,30-33], no comparative study of RNA amplification procedures from picogram amounts has been published.

## Results

**Figure 1:**
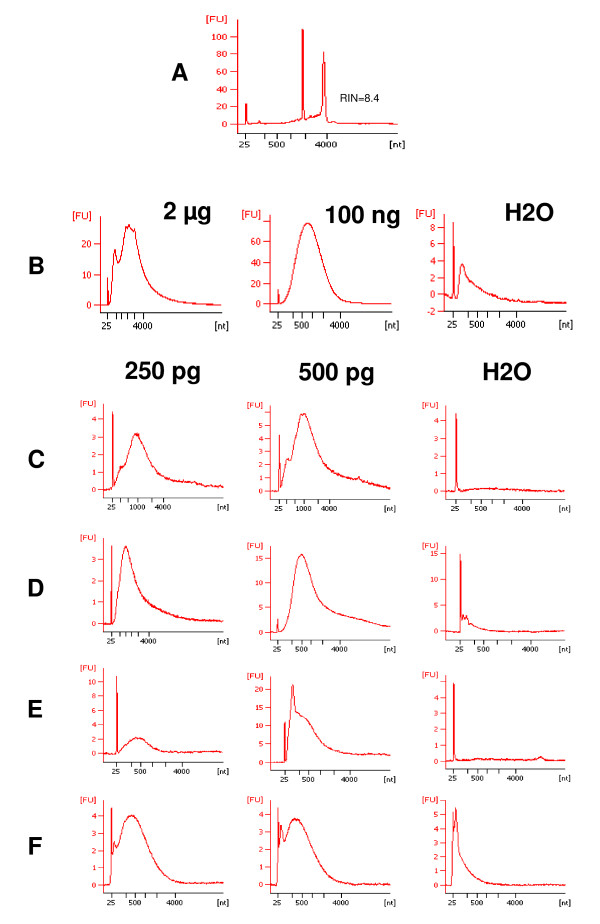
**Quality of input RNA and targets synthesised from different amounts of input RNA**. **A**, Bioanalyzer electrophoretic profile of the diluted Universal Human Reference RNA used as input for all amplifications. This profile corresponds to a classical and non-degraded human RNA with two fine characteristic peaks corresponding to 18S and 28S RNAs. 8.4 corresponds to the RNA Integrity Number (RIN) and reflects the high quality of this RNA **B**, electrophoretic profiles of cRNA obtained after one- and two-round Affymetrix amplification using 2 μg, and 100 ng of input RNA respectively or water as negative control. **C**, **D**, **E**, **F**, electrophoretic profiles of cRNA or cDNA obtained using Ambion (**C**), Arcturus (**D**), Epicentre (**E**) and Nugen (**F**) amplification systems from 250 pg and 500 pg of input RNA, or water as negative control.

Qualitative and quantitative analysis of the input total RNA confirmed its high quality (Figure 1A).

### Qualitative and quantitative analysis of amplification products

For each protocol, one RNA amplification was performed from 250 pg and one from 500 pg of human universal RNA by two operators in two independent laboratories. A negative control (amplification without total RNA) and a positive control (if available) were included in each experimental batch. The quality of each amplified product (cRNA or cDNA) was assessed using microfluidic electrophoresis (Figure 1C–F) and compared to the amplified aRNA obtained from 2 μg and 100 ng RNA inputs following the standard protocol proposed by Affymetrix (Figure 1B).

The size of the main population of aRNAs synthesised according the protocol proposed by Ambion (Figure 1C) ranged between 100 nt and 4000 nt with an average of 1000 nt which is shorter than the manufacturer's indicated length. Moreover, an unexpected short-sized aRNA population of 200 nt was consistently observed. The population of aRNA obtained using the Arcturus protocol ranged in size between 100 and 2000 nucleotides (nt) with an average of 500 nt, but aRNAs longer than 8000 nt were systematically observed (Figure 1D). The aRNA population generated according to the Epicentre TargetAmp™ protocol (Figure 1E) ranged in size between 25 nt and 1000 nt, with an average of 200 nt, which is shorter than indicated in the manufacturer's specifications. The electrophoretic traces of the aRNA obtained during the duplicate amplifications of 250 pg and 500 pg of starting total RNA were also different. The size of cDNAs obtained using the amplification method proposed by Nugen (Figure 1F) was distributed between 100 nt and 2000 nt (average: 500 nt) according to the manufacturer's protocol. However, a discrete population cDNA of 100 nt was systematically observed, clearly corresponding to non-specific products that were also detected on amplification of the negative control.

The quantities of aRNA and cDNA obtained after amplification are presented in Table 1. Yields were sufficient to perform target preparation and hybridisation according to the manufacturers' protocols except for amplification performed using Epicentre TargetAmp™ system. Moreover, yields obtained with the protocols proposed by Epicentre and Arcturus appeared to be laboratory-dependent and non-reproducible in our hands (twofold changes in the amounts of amplified cDNA). Regardless of the protocol used, similar yields of about 2 μg of non-specific amplification products were synthesised when amplification was performed without RNA (negative controls).

**Table 1:** aRNA and cDNA yields obtained after amplification using universal RNA, positive and/or negative controls.

**Protocol**	**Sample**	**Starting amount**	**Amount of amplified material**
Ambion	Universal RNA	500 pg	88.1 μg
			73.9 μg
	Universal RNA	250 pg	65.1 μg
			50.5 μg
	Negative control	0 pg	2.5 μg

Arcturus	Universal RNA	500 pg	34.3 μg
			15.3 μg
	Universal RNA	250 pg	45.6 μg
			16.2 μg
	Negative control	0 pg	1.9 μg
	Positive control RNA	500 pg	24.3 μg
			19.7 μg

Epicentre	Universal RNA	500 pg	55.6 μg
			6.3 μg
	Universal RNA	250 pg	9.4 μg
	Negative control	0 pg	1.9 μg
	Positive control RNA	500 pg	5.1 μg
			7.4 μg

Nugen	Universal RNA	500 pg	7.6 μg
			7.4 μg
	Universal RNA	250 pg	6.9 μg
			8.3 μg
	Negative control	0 pg	1.8 μg

For each protocol, one RNA amplification was performed from 250 pg and one from 500 pg of human universal RNA by two operators in two independent laboratories. Arcturus and Epicentre kits contain an internal positive control RNA. These RNA were systematically included when performing experiments with those chemistries. Negative control experiments (amplification without total RNA) were also performed to evaluate template-independent nucleic acid synthesis.

### Overall quality of expression profiles

After amplification, each labelled cRNA or cDNA target was biotinylated, fragmented and hybridised on Affymetrix HG U133 plus 2.0 to assess the impact of each protocol on expression profiles. Raw .CEL files were normalised using the MAS5 algorithm from Affymetrix, and metrics for all hybridisations were analysed and compared (Table 2). Targets synthesised with each of the methods assessed showed acceptable average background values compared to Affymetrix specifications, ranging between 20 and 100. Background values of chips hybridised with cDNA targets (Nugen WT-Amplification™) were homogeneous and significantly lower than those of the other chips. Percentage of present calls (%P, i.e. expressed genes) ranged from 40% to 50%, as expected when analysing human universal RNA, except for the hybridisation performed from 500 pg of input RNA according to the Arcturus RiboAmp™ method (about 30% of present calls). Biases toward amplification of 3' and 5' ends of GAPDH and β-Actin were also computed (Table 2) in order to evaluate the efficiencies of cDNA template synthesis and amplification reactions. The 3'/5' ratios for housekeeping genes should be at most 3 when one-round amplification is performed, and at most 10 for two-round amplification procedures. The 3'/5' ratios obtained were dramatically above the cut-off values when targets were synthesised using the protocols proposed by Arcturus, Ambion, and Epicentre but remained acceptable (below 10) for cDNA targets prepared according to the Nugen method.

**Table 2:** Comparison of hybridisation quality metrics of the evaluated RNA inputs and protocols.

**Protocol**	**RNA input**	**Background**	**Present Call**	**β-Actin 3'/5' ratio**	**GAPDH 3'/5' ratio**
Ambion	500 pg	58.4	42%	139.1	11.2
		32.0	48%	235.5	6.5
	
	250 pg	58.4	38%	215.6	13.5
		32.4	44%	123.3	7.6

Arcturus	500 pg	54.0	29%	96.3	25.2
		52.4	30%	70.5	15.0
	
	250 pg	32.7	37%	36.5	14.2
		43.9	41%	33.3	9.4

Epicentre	500 pg	58.4	43%	30.9	11.4
		-	-	-	-
	
	250 pg	-	-	-	-
		-	-	-	-

Nugen	500 pg	33.5	45%	6.6	1.1
		35.7	49%	7.2	1.2
	
	250 pg	31.5	39%	6.9	0.8
		32.8	45%	10.6	1.3

Affymetrix, One-round amplification protocol	2 μg	44.9 44.7	50% 49%	1.5 2.6	1.0 1.2

Affymetrix, Two-rounds amplification protocol	100 ng	66.7 54.7	52% 47%	12.2 12.8	1.3 1.8

'-': no hybridisation performed due to poor yield after amplification.

Targets were synthesised, in each laboratory (see Table 1), with the five protocols and hybridised to HG U133 Plus 2.0 array. A rapid analysis was performed with Expression Console (Affymetrix, MAS 5 normalisation).

### Reproducibility and inter-system comparability

**Figure 2:**
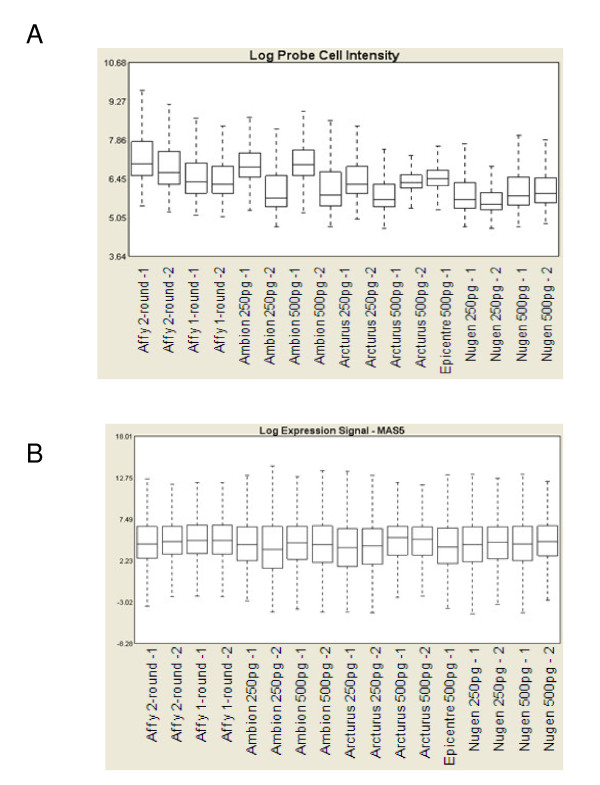
**Box plots of signal intensities**. **A**, raw data. **B**, data after MAS5 normalisation.

Raw signal distributions before (Figure 2A) and after normalisation (Figure 2B) were analysed and compared. After normalisation, the signal distributions of chips hybridised with all protocols were homogeneous and can be compared. Note that the results obtained, before normalisation, with RNA amplified according to the Ambion MessageAmp™ protocol highlight the discrepancies in signal distribution that appear to be batch- or laboratory-dependent.

**Figure 3:**
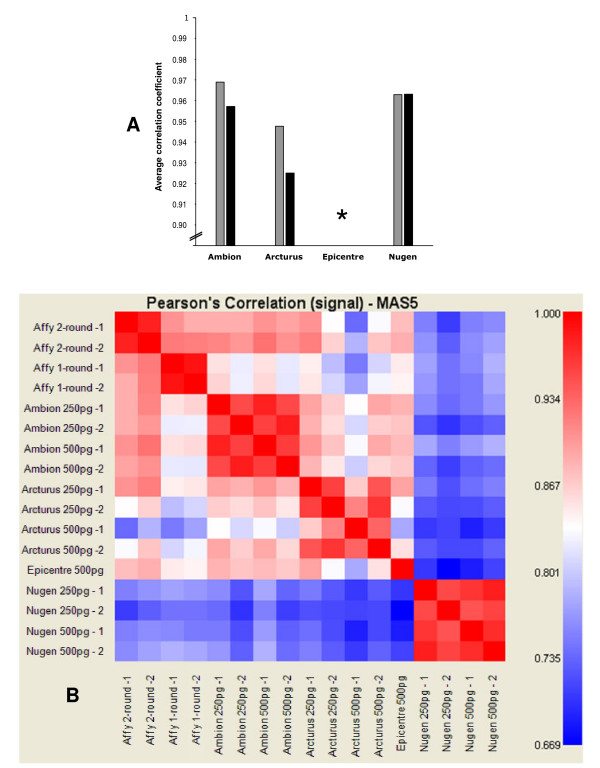
**Statistical analysis of expression level measurements for assessing reproducibility and comparability of amplification chemistries**. **A**, we calculated the Pearson's correlation coefficients between technical replicates (same chemistry, same amount of input RNA, but different laboratories). The correlation values were then averaged for each chemistry (grey bars). We also calculated correlation coefficients between results obtained from 250 pg and from 500 pg of RNA input (same chemistry, but different amount of RNA input) and averaged them for each chemistry in order to evaluate the robustness across quantitative variability of RNA input (black bars). Most Epicentre amplifications did not yield sufficient aRNA to carry out hybridisations, and Pearson's correlation coefficients could therefore not be calculated (*). **B**, Graphic representation of Pearson's correlation coefficients calculated for each pairwise comparison of all assays.

To assess the overall reproducibility of amplifications, Pearson's correlation coefficients between technical replicates were computed and averaged for each protocol (Figure 3A). The average of correlation coefficients for all protocols was about 0.95 indicating high reproducibility of measurements. Correlation coefficients between results obtained from 250 pg and 500 pg of RNA input were also averaged for each method to evaluate protocol reproducibility and robustness across quantitative modulations of RNA inputs (Figure 3A). Almost no difference was observed for correlations between technical replicates and correlations between 250 pg and 500 pg of RNA input for the Ambion and Nugen protocols, suggesting that the amount of RNA input has little impact on expression data. A lower correlation coefficient (0.924) was observed between the results obtained with different amounts of RNA input when using the Arcturus RiboAmp™ protocol.

The correlation of the gene expression data obtained with each evaluated methods and the reference one-round and two-round Affymetrix amplification protocols were also compared to assess the value of each protocol (Figure 3B). Not surprisingly, reproducibility within a given protocol was higher than across protocols. Major discrepancies between hybridisations of cDNA synthesised according to the Nugen WT-Amplification™ protocol and aRNA produced according to the IVT amplification protocols (protocols proposed by Affymetrix, Ambion, Arcturus, and Epicentre; Pearson's correlation coefficient of about 0.7) were observed. Moreover, more marked discrepancies were observed between data from Affymetrix one-round IVT amplification and data from all two-round IVT amplifications, than between each of the two-round IVT amplifications. Not surprisingly, the most strongly correlated results were obtained using similar amplification systems.

### Further assessment of the Nugen protocol

**Figure 4:**
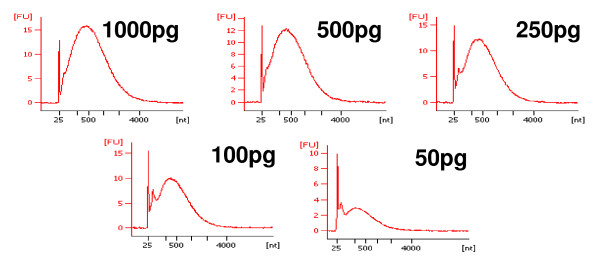
**Quality of cDNA targets synthesised following Nugen protocol from different amounts of input RNA**. Bioanalyzer electrophoretic profiles of cDNA targets obtained after the amplification of 50 pg, 100 pg, 250 pg, 500 pg, and 1 ng of total RNA using Nugen chemistry.

**Figure 5:**
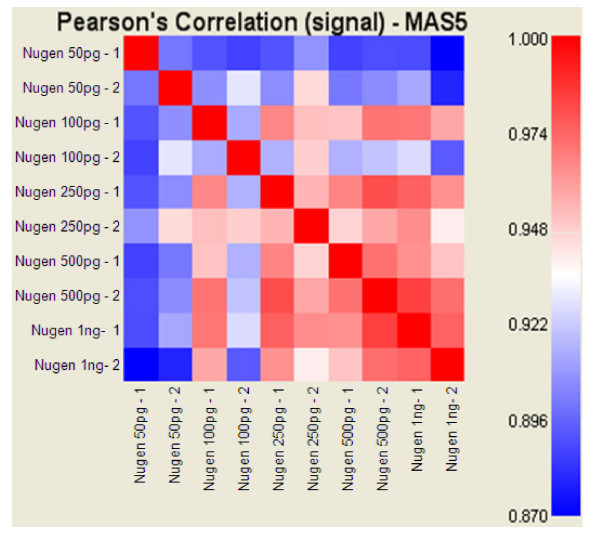
**Reproducibility of expression data obtained using Nugen chemistry across amounts of input RNA**. Pearson's correlation coefficients were computed and represented graphically for each pairwise comparison of assays. Lower correlations were observed for data obtained from smallest amounts of input RNA (50 pg and 100 pg).

The Nugen WT-Ovation™ pico system was further characterised, as the overall best results were obtained using this protocol. Additional amplifications and microarray hybridisations from 50 pg, 100 pg and 1000 pg of input RNA were performed in duplicate as previously. The amounts of input RNA evaluated therefore ranged from 50 pg to 1 ng (50 pg, 100 pg, 250 pg, 500 pg, and 1000 pg). The quality of each amplified cDNA was assessed (Figure 4). Not surprisingly, the discrete peak corresponding to non-specific amplification products previously observed in negative controls, increased relative to the specific cDNA target quantity when the amount of input RNA decreased. Amplification yields (Table 3) decreased slightly with the amount of input RNA. Except for the amplifications performed from 50 pg of input RNA, more cDNA was generated than required for GeneChip hybridisation according to the Nugen WT-Amplification™ pico protocol (5 μg, Table 3). Amplified cDNA products, including those containing less than 5 μg, were biotinylated, fragmented and hybridised on Affymetrix HG U133 plus 2.0 under the same experimental conditions as those described previously. Hybridisation metrics were analysed and compared as described previously (Table 3). Average background values were low and homogeneous with those obtained previously with 250 pg and 500 pg of input RNA. The other metrics were within the expected range according to the usual specifications for amplifications of 1000 pg, 500 pg and 250 pg, but the percentages of present calls decreased for amplifications of 100 pg and 50 pg of RNA, and 3'/5' ratios for housekeeping genes were significantly higher for amplifications performed from 50 pg of RNA (Table 3). To further investigate the impact of RNA input amounts on expression data, Pearson's correlation coefficients between all experimental data were computed (Figure 5). A weaker correlation was observed for data obtained from the smallest amounts of input RNA (50 pg and 100 pg).

**Table 3:** cDNA yields and hybridisation quality metrics of Nugen experiments.

**RNA input**	**Amount of amplified cDNA**	**Background**	**Present Call**	**β-Actin 3'/5' ratio**	**GAPDH 3'/5' ratio**
1000 pg	10.6 μg	31.9	45.6%	8.7	1.3
	6.1 μg	29.7	52.1%	6.0	1.0

500 pg	7.6 μg	33.5	45.1%	6.6	1.1
	7.4 μg	35.7	48.6%	7.2	1.2

250 pg	6.9 μg	31.5	39.3%	6.9	0.8
	8.3 μg	32.8	45.5%	10.6	1.3

100 pg	6.1 μg	29.8	37.8%	10.5	1.4
	4.4 μg	32.4	29.4%	6.2	1.0

50 pg	3.5 μg	30.7	19.6%	23.6	1.5
	4.5 μg	30.0	25.4%	11.1	1.2

0 pg	1.8 μg	-	-	-	-

Targets were synthesised in each laboratory with the Nugen protocol at five amount of input RNA and hybridised to HG U133 Plus 2.0 array. A rapid analysis was performed with Expression Console (Affymetrix, MAS 5 normalisation).

**Figure 6:**
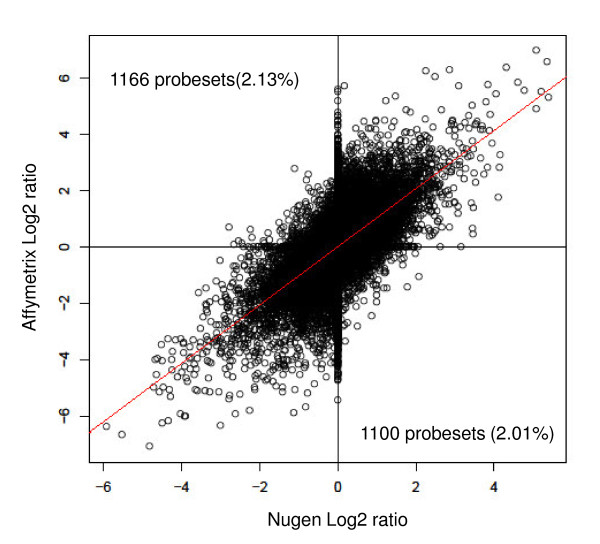
**Comparison of differential gene expression data using Affymetrix one-round and Nugen chemistries**. Targets were generated from RNAs of two cell lines using Affymetrix 1-round IVT and Nugen chemistries. After hybridisation, differential gene expression results were normalised (MAS5) and the expression ratio calculated for each probeset. Logarithm of ratios to the base 2 (Log2 ratios) were computed for each chemistries and graphed(X axis Nugen and Y axis Affymetrix). Differences in the direction of differential expression were only observed for 4% of the probesets (dots located in the upper-left quadrant and lower-right quadrant). Moreover, almost all of those probesets show a low differential expression (absolute value lower than 1, less than 2-fold over or lower expression). The slope of the regression line is 1.033 (p < 10^-16^) and it intercepts the Y axis at -0.02 (p < 10^-12^). The Pearson's correlation coefficient has been calculated between the expression ratios distribution (R = 0.321).

To assess the sensitivity of Nugen system and to determine whether it allowed the measurement of differential gene expression, the expression profiles of two cell lines (SkBr3 and HCC38) were compared using the Nugen WT-Amplification™ pico system and the Affymetrix one-cycle IVT amplification. 500 pg and 2 μg of RNA of each cell line were amplified according to the protocols proposed by Nugen and Affymetrix, respectively, and hybridised on Affymetrix HG U133 plus 2.0 GeneChip. As expected, Affymetrix and Nugen protocols showed similar yields of reproducibility with correlation coefficients up to 0.90 between replicates (data not shown). Differential gene expression analysis was performed for each system using the present, absent, marginal increase, marginal decrease, increase and decrease criteria proposed by Affymetrix and implemented in GCOS software (Table 4). Although the two systems allowed identification of the same number of up- or down-regulated genes, only about 50% of these genes were differentially expressed in the same direction when using the other protocol. The other set of genes was mostly non-modulated. The direction of differential expression was divergent in only 0.3% of measurements between the Nugen WT-Amplification™ pico system and the Affymetrix one-round IVT amplification system. Another evaluation of the differential gene expression measurement has been performed. For each chemistry, ratios of the expression values obtained for each cell line were calculated and graphed (Figure 6). Although ratios show dispersion with respect to the chemistry, no particular bias was noticed. In addition, differences in the direction of differential expression were observed for 4% of the probesets (1100 over-expressed probesets with Nugen and down-expressed with Affymetrix were observed and inversely for 1166). Moreover, almost all of those probesets show a low differential expression (absolute value lower than 1, less than 2-fold over or lower expression).

**Table 4:** Comparison of differential gene expression data using Affymetrix one-round and Nugen chemistries.

**Affymetrix**			**Nugen**		
**status**	**# probsets**	**% probsets**	**status**	**# probsets**	**% probsets**

D	7423	13.6%	D	3908	52.6%
			MD	127	1.7%
			NC	3262	43.9%
			MI	11	0.1%
			I	115	1.5%

I	7057	12.9%	D	140	2.0%
			MD	12	0.2%
			NC	2996	42.5%
			MI	118	1.7%
			I	3791	53.7%

MI	268	0.5%	D	11	4.1%
			MD	1	0.4%
			NC	168	62.7%
			MI	4	1.5%
			I	84	31.3%

MD	317	0.6%	D	94	29.7%
			MD	5	1.6%
			NC	203	64.0%
			MI	1	0.3%
			I	14	4.4%

NC	39610	72.4%	D	3358	8.5%
			MD	268	0.7%
			NC	32811	82.8%
			MI	224	0.6%
			I	2949	7.4%

Targets were generated from RNAs of two cell lines using Affymetrix 1-round IVT and Nugen chemistries. After hybridisation, differential gene expression results were analysed. Status is defined as Decrease (D), Increase (I), Marginal Increase (MI), Marginal Increase (MI), and No Change (NC).

## Discussion

Gene expression profiling on several hundred cells isolated by cell-sorting technologies, or by LCM prompts the development of new amplification procedures. Over recent years, academic, private sector and commercial researchers have been developing tools and methods to generate expression profiles from small amounts of biological material. Improvements have been made in all steps of the process of expression profiling experiments such as sample collection, RNA purification, or acquisition and analysis of expression data. Over the last few years, improvements in RNA amplification methods now allow the synthesis of sufficient targets to perform microarray hybridisation on as little as picogram amounts of input RNA. In this study, we evaluated and compared four commercial RNA amplification protocols using picogram amounts of input RNA. This comparative study will be useful to researchers when planning new experiments involving samples derived from a few cells.

This comparative study was based on several assessments of amplification products. First of all, electrophoresis was performed and examined in combination with cRNA or cDNA yields. This analysis is performed routinely prior to hybridisation, as it provides an estimate of the overall amplification performance. Unusual traces or insufficient yield correspond to either abnormal input RNA, or amplification failure. The poor yield obtained with the protocol proposed by Epicentre is due to amplification failures since the quality of the commercial RNA has been checked. Experimental errors might be the cause of these failures. However, as amplifications were carried out in duplicate by two experienced operators in two laboratories, the quality of the batch and its reproducibility may be responsible for these errors.

The overall size of aRNA, estimated by the electrophoretic profile, has been previously described as a good metric when looking at within-sample fidelity [26]. As expected, a high level of consistency in amplified product sizes and electrophoretic profiles was observed within each technology, since all amplifications were performed using the same input RNA. The repeated observation of products of very large size (8,000–12,000 nt) obtained with the Arcturus RiboAmp™ kit are surprising and might be due to the transcription of non-specific ligation of cDNA templates. As for other microarray technologies, probes designed for Affymetrix gene expression assay are biased towards the 3' end of the transcripts: the length of the target is therefore not a critical metric provided the targets cover the first 700 bases from the 3' end of the transcripts. However some probes (10% at most) represent sequences over 600 bases from the 3' end, and short amplification products may therefore not address these probes. The impact of this issue can be easily estimated by the 3'/5' ratios of housekeeping genes. Affymetrix HG U133 plus 2.0 GeneChips include probe sets selected in the 5' region of housekeeping genes in addition to conventional probes within a maximum of 600 nt from the 3' end. The signal intensity ratio of the 3' probe over the 5' probe (3'/5' ratio) is a good metric to evaluate the qualitative performance of first strand cDNA synthesis and aRNA transcription (or cDNA replication for Nugen WT-Amplification™). Abnormally high ratios were obtained when using T7-IVT-based amplification chemistries suggesting that the 3' biased expression issue should be taken into consideration when analysing these data. The lower 3'/5' ratios observed in the Nugen WT-Amplification™ approach could be explained by the fact that i) in addition to the conventional poly-T priming for the initial reverse transcription, the system proposed by Nugen also includes random priming, and ii) amplification is performed in one cycle thus avoiding shortening of targets at the 5' end by performing a random primed reverse transcription of first-round amplified aRNA.

The percentage of present call has been previously described as a good metric when evaluating the sensitivity of a method [30,34]. Gene expression analysis performed on small amounts of input RNA, can be logically expected to give a reduced sensitivity. However, only Arcturus RiboAmp™ chemistry showed significantly reduced sensitivity compared to the standard procedure. Poor sensitivity was also observed when conducting experiments using Nugen WT-Amplification™ system using the lowest amounts of input RNA (50 pg and 100 pg), suggesting that the minimum input limits of the protocol had been reached.

With respect to hybridisation specificities, the Nugen technology shows a fundamental difference as it generates a single-stranded cDNA target whereas others protocols yields cRNA targets. As RNA/DNA interactions are stronger than DNA/DNA interactions, cDNA hybridisations should theoretically be more specific but also less sensitive. However, the protocol proposed by Nugen was not associated with decreased sensitivity and the lower average background value of the chips hybridised with cDNA targets (Nugen) suggests reduced non-specific target/background interactions but additional studies should be conducted to evaluate the potential increased specificity of the probe/target interaction and its biological impact on expression profiling.

Practical criteria should be considered to choose an amplification approach to carry out expression profiling experiments. These include completion time, handling difficulties and labour intensiveness. Major differences were observed between the various protocols evaluated in terms of these criteria. IVT-based amplifications (Ambion MessageAmp™, Arcturus RiboAmp™, and Epicentre TargetAmp™) involve numerous steps and are therefore time-consuming and labour-intensive. However, the high quality of technical support and optimisation of chemistries (most reagents are conveniently pre-dispensed and pre-mixed) simplify the handling of the Ambion and Arcturus systems. The Nugen technology is fundamentally different from the IVT-based amplifications. Fewer steps are needed to achieve amplification, and the process does not include delicate RNA handling during the amplification process. The Nugen WT-Amplification™ protocol is therefore shorter and easier to complete, and consequently less error-prone. Ultimately, investigators will need to compare experimental results obtained by different laboratories. Experimental variables (such as operator) on expression data could be limited if fewer steps are needed to achieve amplification.

When expression profiling experiments are performed on just a few cells, it is currently impossible to strictly assess the quality and quantity of the purified RNA. Even the most modern and accurate spectrophotometers or microfluidic-based electrophoresis chips require at least 100 pg of RNA to characterise nucleic acids extracted from such minute samples. The results presented here show that a two-fold variation of the amount of input RNA had almost no impact on the transcriptomes analysed when starting with picogram amounts of RNA. Moreover, high reproducibility within a given protocol (correlation coefficient of about 0.95) was observed for amplifications that were carried out in two different laboratories by two different operators. This shows that trained laboratories could conduct amplifications with the technologies proposed by Ambion, Arcturus, or Nugen and produce data that could effectively be compared across laboratories using the same amplification protocols. However, the results obtained in this study indicate large variations between different protocols. This suggests, as previously shown, that i) when conducting expression profiling experiments the same amplification protocol should be used in order to maximize the comparability of the results [26,31,33,35,36], and ii) that RNA amplification affects the expression measurements [35,37-39]. However, researchers who already have a large volume of Affymetrix two-round amplification data, and who want to conduct a project including comparison of these data, might decide to choose the Ambion MessageAmp™ amplification protocol despite its very high bias towards the 3' to 5' ends, as expression profiles estimated according to this protocol exhibited the best correlation with the results of Affymetrix amplification (correlation coefficient of 0.89).

Amplifications were performed by two operators in two different laboratories in order to mimic "real experimental conditions" of expression profiling. In our hands and under these experimental conditions, Nugen WT-Amplification™ pico protocol appeared the most suitable. Additional experiments were performed in order to further assess this system. Amplifications were performed on amounts of input RNA ranging from 50 pg to 1 ng to test the system at the upper and lower limits of input amounts corresponding to a few cells isolated by FACS or LCM. The results obtained show that the overall quality, comparability and reproducibility of expression measurements were very good for RNA input amounts ranging from 250 pg to 1000 pg. The system was less efficient with 100 pg and 50 pg of RNA inputs with respect to sensitivity and reproducibility. It is important to analyse this result in the light of manufacturer's specifications indicating that the minimal RNA input of the system is 500 pg.

The results of a differential gene expression experiment using Nugen WT-Amplification™ chemistry or Affymetrix one-round IVT amplification were also compared. If we strictly count the number of probesets with the same status (Decrease, Increase, Marginal Increase, Marginal Decrease or No Change) in both chimistries, discrepancies were observed only for 25% of probesets. However the direction of differential expression diverged in few measurements. These results show that most genes have similar differentially expressed patterns and that one amplification protocol does not create an artificial variability of the measurements compared to the other. The observed discrepancies do not appear to be due to the decreased sensitivity of one of the two protocols because the same proportion of genes was modulated when using the Affymetrix and Nugen protocols. Although the effects of RNA amplification on differential gene expression measurements have been previously reported [26,35,37-39], a large proportion of these discrepancies might be due to fundamental differences between the two approaches, such as the influence of the molecular nature of the targets (RNA or DNA) on expression measurement, or the use of random priming to synthesise double-stranded cDNA templates.

The preservation of the relative abundance levels of gene transcripts is an important issue when performing RNA amplification prior to genome-wide expression measurements. This issue has been widely studied in comparing expression measurements i) from the amplification of different amounts of RNA [11,21,40], ii) from amplified and unamplified materials [14,21,41,42], iii) using different amplification procedures [11,15,26,43], iv) to a gold standard amplification procedure[26,44], v) using different expression evaluation methods such as quantitative-reverse-transcription-Ploymerase-Chain-Reaction (qRT-PCR) [21,26,36,43]. Most of these studies were performed using IVT-based amplification methods. Those methods were different from the ones evaluated in this report, and were not optimised for picogram amounts of input RNA. Only two studies were conducted using Nugen amplification technology but not the WT-Amplification™ pico system evaluated here [21,26]. The general conclusion that could be drawn from these articles is that either IVT-based or Nugen-like RNA amplifications were globally able to maintain relative transcripts abundance with only slight differences. However, it has been pointed out that, in most of these reports, the statistical analyses or the comparison with qRT-PCR data were restricted only to subsets of genes such as outliers or highly expressed genes [31,37,38]. Furthermore, several studies have seriously questioned the preservation of the relative transcript abundance during RNA amplification. Sequence dependent biases [35,37], and a drop of fidelity for low expressed transcripts [10,39,45,46] have been demonstrated. In addition, it has been shown that low expressed genes were subject to stochastic fluctuations that increase as the sample size decreases [31,38,46]. However, we obtained high correlations between technical replicates using either Ambion, Arcturus or Nugen methods (Figure 3A). It shows that no or negligible stochastic fluctuations occur at these levels of input RNA (500 pg and 250 pg). On the other hand, stochastic event could explain the lower reproducibility observed for smaller amount of input RNA (50 pg and 100 pg) using Nugen method.

Therefore, a) we do not recommend to perform amplification from less than 250 pg of input RNA, and b) when it is possible, researchers should try to increase the RNA input quantity to at least 500 pg to be more confident in the biological interpretation of the results, particularly concerning low express transcripts.

## Conclusion

In this study, we evaluated and compared four commercial RNA amplification protocols using picogram amounts of input RNA. An operational flow chart was designed to evaluate the effects of laboratory and operator intervariability on expression results. In our hands, the WT-Ovation™ pico system proposed by Nugen appears the most suitable for amplification and gene expression analysis. The amplification method proposed by Nugen is fast, easy to perform, and does not require several rounds of amplification or exponential PCR cycles. It provides a high quality expression profile that appears to allow identification of differentially expressed genes. Finally, the reproducibility of the results of amplification across laboratories and across amounts of RNA input indicates that this system is an efficient tool to conduct gene expression profiling experiments from LCM or cell-sorted isolated samples.

## Methods

### RNA preparation and assessment

RNA derived from SkBr3 and HCC38 cells was purified using the RNeasy Mini Kit (Qiagen) including on-column DNase (Qiagen) digestion as described by the manufacturer's protocol. The Universal Human Reference RNA (Stratagene) representing a pool of 10 different human cell lines was used as control RNA.

After nucleic acid quantification using a ND-1000 spectrophotometer (Nanodrop Technologies), all RNAs were serially diluted in RNAse-free water to obtain a 250 pg/μL stock solution. RNA quality was ensured by analysing separation trace of RNA using the RNA6000 PicoAssay for the Bioanalyzer 2100 (Agilent). Aliquots were prepared and stored at -80°C. The same RNA was used for all experiments as starting RNA for amplification. Each aliquot was used once.

When provided within the amplification kits, internal RNA controls were also used as starting material (MessageAmp™ II aRNA Amplification (Ambion), RiboAmp™ HS RNA Amplification Kit (Arcturus)).

### Logistics

All amplification experiments were carried out in duplicate by two trained operators in two different laboratories in order to assess the consistency and robustness of amplification and labelling protocols. Microarray hybridisations, quantitative and qualitative measurements were performed by the same experienced technician on the same instruments to ensure that expression level discrepancies were only due to amplification and labelling procedures.

### Amplification and labelling of small RNA samples

RNA amplifications were performed using the following kits: One- and two-round amplification kit (Affymetrix), RiboAmp™ HS RNA Amplification Kit (Arcturus), MessageAmp™ II aRNA Amplification (Ambion), TargetAmp™ 2-Round Aminoallyl-aRNA Amplification (Epicentre), and WT-Amplification™ Pico (NuGEN). For all experiments, the manufacturers' protocols were strictly followed.

Briefly, the standard protocol described by Affymetrix amplifies and produces after a single round T7 amplification, more than 40 μg of labelled cRNA from 1–4 micrograms of total RNA. Total RNA is used for cDNA synthesis using an oligo-dT-T7 primer. The cDNA is used as template for double-strand DNA (dsDNA) synthesis and purification. Linear amplification and labelling of complementary RNA (cRNA) is performed by *in-vitro *transcription. When input RNA quantity is below standard requirements, a two-round amplification protocol is performed with 10–100 ng of total RNA. In this case, the first IVT does not use any labelled nucleotide to generate unlabelled cRNA. This cRNA is used for reverse transcription (RT) with random primers, dsDNA synthesis and finally labelled cRNA synthesis.

The MessageAmp™ II aRNA Amplification Kit (Ambion) is based on the RNA amplification protocol described by Eberwine et al. [6], and designed to generate sufficient material to hybridise an Affymetrix Genechip. The procedure to synthesise cRNA is similar to the Affymetrix two-round amplification and consists of RT with oligo(dT) primer bearing a T7 promoter followed by dsDNA and unlabelled cRNA synthesis. A second round of amplification is performed with random primers to generate DNA. The sense DNA then undergoes second strand synthesis and clean-up to become a template for the second *in vitro *transcription (IVT) using labelled nucleotides.

RiboAmp HS version C (Arcturus RiboAmp HS protocol) enables target synthesis from 100 pg to 500 ng. Version C of RiboAmp HS is composed of two-round amplification similar to the protocols proposed by Affymetrix or Ambion. Reactions were performed according to the manufacturer's protocol.

The TargetAmp 2-Round Aminoallyl-aRNA Amplification Kit 1.0 (Epicentre) is designed to be an improvement of the other linear T7 RNA amplification process described above to produce microgram amounts of aminoallyl-aRNA from as little as 10 pg of total RNA. Two rounds of amplification combine two generations of SuperScript Reverse Transcriptases from Invitrogen.

In contrast with other manufacturer protocols, the WT-Ovation™ Pico RNA Amplification System (Nugen) is not based on T7 polymerase cRNA synthesis. Nugen has designed a technique called Ribo-SPIA™, which is a three-step process that generates amplified cDNA from as little as 500 picograms of total RNA[21]. First strand cDNA is prepared from total RNA using a unique first strand DNA/RNA chimeric primer mix. The primers have a DNA portion that hybridises either to the 5' portion of the poly(A) sequence or randomly across the transcript. Reverse transcriptase extends the 3' DNA end of each primer generating first strand cDNA/mRNA hybrid. Second strand cDNA synthesis step generates double stranded products with RNA-DNA heteroduplex at one end. The third step is the DNA amplification, called SPIA™ amplification using a specific DNA/RNA chimeric primer, DNA polymerase and RNase H in a homogeneous isothermal assay that provides highly efficient amplification of DNA sequences. RNase H is used to degrade RNA in the DNA/RNA heteroduplex at the 5' end of the first cDNA strand. This results in the exposure of a DNA sequence that is available for binding a second SPIA™ DNA/RNA chimeric primer. DNA polymerase then initiates replication at the 3' end of the primer, displacing the existing forward strand. The RNA portion at the 5' end of the newly synthesised strand is again removed by RNase H, exposing part of the unique priming site for initiation of the next round of cDNA synthesis. The process of SPIA™ DNA/RNA primer binding, DNA replication, strand displacement and RNA cleavage is repeated, resulting in rapid accumulation of cDNA with a sequence complementary to the original mRNA.

The labeled cRNA targets are synthesised using the genechip IVT labelling kit (Affymetrix) from dsDNA obtained with Affymetrix and Arcturus protocols. TargetAmp amplification products (Epicentre protocol) are labelled using the Biotin-X-X-NHS kit (Epicentre). WT-Ovation™ Pico products (NuGEN) are labelled using the FL-Ovation™ cDNA Biotin Module V2 (NuGEN). Each labeled cRNA targets are synthesised according to manufacturer's protocols.

### Amplification products quantification and quality control

The quantity and quality of the amplified cRNA or cDNA were assessed by a ND-1000 spectrophotometer (Nanodrop Technologies), Agilent Bioanalyzer and RNA 6000 nanoChips (Agilent Technologies, Santa Clara, CA), respectively.

### Microarray hybridisation

Hybridisation mixtures were prepared according to Affymetrix procedures to accommodate 10–20 μg of labelled cRNA targets from Affymetrix, Arcturus, Ambion and Epicentre amplifications, or 5 μg of cDNA targets from NuGEN amplification. If less than 10 μg of cRNA or 5 μg of cDNA targets were generated, the entire quantity of material was used as input. Human Genome U133 Plus 2.0 GeneChips (Affymetrix) were hybridised, revealed and washed according to the Affymetrix protocol. GeneChips were scanned using a 7 G scanner (Affymetrix) and images (DAT files) were converted to CEL files using GCOS software (Affymetrix). All of the microarray raw data have been deposited in the Gene Expression Omnibus under the accession number GSE15398 

### Data Analysis

Data analysis was performed using expression console V 1.1 (Affymetrix) and genome analysis tools (Partek). Differential gene expression analysis was performed using GCOS software (Affymetrix).

## Authors' contributions

MCZ, CB and CD designed the study. MCZ and DG performed the experiments. MCZ, DG and CD performed the data analysis. MCZ, DG and CD drafted the manuscript. CB, JPT, SL and CD supervised the study. All authors read and approved the final manuscript.
